# Time Course of Neurobehavioral Disruptions and Regional Brain Metabolism Changes in the Rotenone Mice Model of Parkinson’s Disease

**DOI:** 10.3390/biomedicines10020466

**Published:** 2022-02-16

**Authors:** Dmitry Troshev, Dmitry Voronkov, Anastasia Pavlova, Denis Abaimov, Alexander Latanov, Tatiana Fedorova, Daniil Berezhnoy

**Affiliations:** 1 Koltzov Institute of Developmental Biology, Russian Academy of Sciences, Vavilov Street, 26, 119334 Moscow, Russia; dmitry.vad.troshev@gmail.com; 2 Research Center of Neurology, Laboratory of Clinical and Experimental Neurochemistry, Volokolamskoeshosse, 80, 125367 Moscow, Russia; voronkovdm@gmail.com (D.V.); abaidenis@yandex.ru (D.A.); tnf51@bk.ru (T.F.); 3 Biological Faculty, Moscow State University, Leninskie Gory, 1s12, 119234 Moscow, Russia; pav_nastasya@mail.ru (A.P.); latanov.msu@gmail.com (A.L.)

**Keywords:** Parkinson’s disease model, rotenone, C57BL/6 mice, behavior, learning, neurodegeneration, catecholamines, cytochrome-C oxidase

## Abstract

Parkinson’s disease (PD) is characterized by slow progression with a long prodromal stage and the gradual evolution of both neuropsychological symptoms and subtle motor changes, preceding motor dysfunction. Thus, in order for animal models of PD to be valid, they should reproduce these characteristics of the disease. One of such models, in which neuropathology is induced by chronic injections of low doses of mitochondrial toxin rotenone, is well established in rats. However, data on this model adapted to mice remain controversial. We have designed the study to describe the timecourse of motor and non-motor symptoms during chronic subcutaneous administration of rotenone (4 mg/kg daily for 35 days) in C57BL/6 mice. We characterize the underlying neuropathological processes (dopaminergic neuron degeneration, regional brain metabolism, monoamine neurotransmitter and lipid peroxidation changes) at different timepoints: 1 day, 2 weeks and 5 weeks of daily rotenone exposure. Based on the behavioral data, we can describe three stages of pathology: cognitive changes from week 2 of rotenone exposure, subtle motor changes in week 3–4 and motor dysfunction starting roughly from week 4. Neuropathological changes in this model include a general decrease in COX activity in different areas of the brain (acute effect of rotenone) and a more specific decrease in midbrain (chronic effect), followed by significant neurodegeneration in SNpc but not VTA by the 5th week of rotenone exposure. However, we were unable to find changes in the level of monoamine neurotransmitters neither in the striatum nor in the cortex, nor in the level of lipid peroxidation in the brainstem. Thus, the gradual progression of pathology in this model is linked with metabolic changes, rather than with oxidative stress or tonic neurotransmitter release levels. Overall, this study supports the idea that a low-dose rotenone mouse model can also reproduce different stages of PD as well as rats.

## 1. Introduction

Parkinson’s disease (PD) is a chronic progressive neurodegenerative disease, affecting about 2–3% of the population over 65 years old [1,2]. It is characterized by the triad of motor symptoms—rigidity, bradykinesia and tremor—becoming progressively worse as the disease advances [3]. Aside from motor symptoms, which are seen after 60% of dopaminergic cells in substantia nigra pars compacta (SNpc) are dead and the level of dopamine in striatum is decreased by more than 70% [4,5,6], PD is characterized by a plethora of neuropsychological symptoms—from depression and anxiety to cognitive decline—which usually manifest earlier in the disease’s progression [7]. Most of these symptoms are still to be matched with the corresponding neuropathological mechanisms. Overall, the progression of PD demonstrates complex temporal dynamics both from the point of behavioral symptoms and underlying neuropathological cascades. Despite several factors that are found to contribute to its onset and progression, from genetic predisposition to environmental toxin exposure [8,9,10], the pathogenesis of sporadic PD remains largely unknown.

To clarify the role of different neurochemical cascades in the mechanism of neurodegeneration in PD and to search for disease-modifying treatments, there is a need for experimental models, reproducing the slow timecourse of PD and its key neurological features. Currently, there area vast number of in vivo PD models used in preclinical research: genetically modified mice, chemical lesions or environmental toxin exposure in rodents and primates [10,11]. However, the majority of murine models of PD are focused on single timepoints of the disease, either in the early or in the late stages of its progression. For example, the use of 6-OHDA or acute high doses of MPTP causes high levels of SNpc dopaminergic cell death within the first few days following injection [4]. Genetic mice models (DJ-1, Parkin and PINK1 knock-outs) are more suitable for the study of pre-motor symptoms, as they usually demonstrate low levels of neurodegeneration [4,12]. There are only several models focused on the chronic progression of the disease, which are suitable for the study of both the pre-motor and motor stages. Most of them utilize either genetically modified mice and chronic exposure of animals to environmental toxins [4,10,13]. One of these models is based on the chronic exposure of rodents to the mitochondrial toxin rotenone [14,15,16], which is a potent inhibitor of NADH-oxidase. This toxin draws special attention since the link between pesticide exposure and PD development has been shown in human epidemiological studies [17]. Reports on rodent models with the use of rotenone confirmed the anatomical, neurochemical and behavioral features of PD and its relatively long disease progression period. However, most of the rotenone effects were studied in rats [14,15], which makes the comparison with novel genetic models, mostly utilizing C57BL/6 mice [4], difficult. At this time, there are several developed toxic models of PD in C57BL/6 mice, utilizing different regime of rotenone exposure; however, they give somewhat controversial results [18,19,20,21,22]. As the rotenone affects not only the nigrostriatal dopaminergic system, but also other brain structures and peripheral tissues, increased mortality and different outcome in the murine experiments is linked with this lack of specificity. Due to this, new models, utilizing chronic low-dose rotenone exposure, attract much attention [21]. Chronic subcutaneous injection of rotenone tends to be a perspective method [18,21], as it allows to reproduce motor symptoms of PD with much smaller concentrations than oral/intragastric administration of the toxin [19,22]. However, even with subcutaneous administration of rotenone, motor symptoms are not always accompanied by the loss of cells in SNpc [18], which raises the question of underlying neuropathology. In our previous work with the same rotenone model [18], we have shown that the rotenone metabolic effect in the brains of mice is less specific than that of rats, disrupting COX activity not only in the SN, but also in various cortical structures [23]. The comparison of rotenone chronic exposure to CD-1 mice with exposure to another toxin, MPTP, showed faster dynamics of motor symptoms evolution in the rotenone model, but they lacked the specific neuropathological changes that are characteristic of PD [24]. However, the question of whether these effects would be reproduced in the C57BL/6 mice strain, which is more frequently used in PD research, remained open. Thus, the aim of the present study was to describe the timecourse of motor and non-motor symptoms during chronic rotenone administration in C57BL/6 mice and characterize the underlying neuropathological processes at different stages.

## 2. Materials and Methods

### 2.1. Animals and Experimental Procedures

Experiments were conducted on male C57BL/6 mice (*n* = 45) aged between 2 and 3 months, weighing between 25 and 30 g. Animals were kept in standard conditions at a temperature between 22 and 24 °C and maintained a 12:12 h day-night cycle. During the experiment, animals were given food and water ad libitum except the days leading up to the food-motivated motor activity test and food-consumption measurements. All experiments were conducted according to the international standards and bio-ethical regulatory requirements (Guide for the Care and Use of Laboratory Animals–Russian Version, National Academy Press, Washington, DC, USA, 1996) [25]. The protocol of the animal study was approved by the Bioethical Commission of Moscow State University (protocol 82.0 from 8 June 2017) and all measures were taken to reduce the number of animals used.

This study tried to describe the behavioral and neuropathological effects in C57BL/6 mice over the time period of chronic administration of rotenone, a mitochondrial toxin used to model parkinsonism in rodents. Experimental groups of animals (“Rot”, *n* = 36) received daily subcutaneous rotenone injections (4 mg/kg) for 35 days. Rotenone was prepared with sterile vegetable oil containing 2% DMSO. The control group (*n* = 9) received daily injections of a vehicle solution in a volume equal to that of the previous three groups over the course of the entire experiment.

In order to collect biological material (samples), a portion of the animals from the experimental group (“Rot”) were euthanized during the experiment: on the 1st day, 24 h after a single rotenone injection (“Rot 1 d”, *n* = 6), after 2 weeks (“Rot 2 w”, *n* = 9) and after 5 weeks (“Rot 5 w”, *n* = 9). The control group animals (*n* = 9) were euthanized after 5 weeks, together with the animals in the “Rot 5 w” group.

### 2.2. Complex Evaluation of Animal Behavior

To assess the well-being, motor and non-motor symptoms in experimental animals in real-time we used the test procedures detailed in Table 1.

*Monitoring of weight and modified neurological severity scale (mNSS)*. To monitor animals’ well-being and overall state, we monitored their weight daily before the injection. Evaluation of their neurological status was conducted every other day with the use of mNSS [26], adding the modified stepping test [27] (steps per 10 s on a homecage lid: 0 points—more than 5 steps; 1 point—less than 5 steps; 2 points—no steps) and qualitative evaluation of ptosis (0 points—no lid ptosis; 1 point—partial ptosis; 2 points—complete ptosis). The maximum total points—sum for all of the tests—equaled 16, where higher point scores reflect the increase in neurological symptoms. We also used two more specific tests to quantitatively assess animal motor symptoms: the catalepsy test and the elevated grid walking test.

*Catalepsy test.* Catalepsy response was determined with the use of the block test. The mice were placed with their forelimbs on a wooden block (4 cm high), and their latency to descend, i.e., the time during which the animals maintained this position, was measured for a maximum of 30 s.

*Elevated grid walking test.* The test was performed on a 20 × 20 cm grid with 1.5 cm distance between the rods. The grid was placed at a 45-degree angle on a horizontal surface with the upper end of the grid attached to the home cage, so the animals were ascending the grid to get back to their homecage. Pre-trained mice were placed on the lower rods of the grid.

During the climb of the animal along the elevated grid, the following parameters were assessed: the time of the climb (the end of the test is the moment when all the animal’s paws had touched the grid of the home cage, maximum time was capped at 90 s) and the number of mistakes made by the animal during the test session. The paw slips from the grid and positioning of paws between rods were considered mistakes. Prior to testing, mice were trained to climb the grid for two sessions (maximum time was capped at 180 s).

*Open field.* The locomotor activity was investigated in an open field. The open field, a plexiglass box (50 × 50 × 30 cm), was connected to a video system (EthoStudio, Russia). The mice were initially placed into the center of the open field and then the activity was monitored for 5 min. For determination of the horizontal activity, the animal’s path was analyzed and the overall track length in cm was measured. Furthermore, the overall time of freezing and grooming episodes were determined.

### 2.3. Dynamics of the Food-Getting Behavior Formation in the ShuttleBox Test

In this investigation, an original method of operant learning used in our previous study with rats [28] was adapted for mice. Mice feeding behavior was registered in a plexiglass shuttlebox with a smooth black surface, consisting of two identical 25 × 25 × 30 cm compartments equipped with two automatic feeders for rationed food release (one per compartment). Small sugar beads (d = 1 mm) were used as reinforcement. Each session, mice were placed in the right compartment of the box and could shuttle freely between the compartments for 5 min. After the first approach of the animal to any of the feeders, it received a single reinforcement. The second reinforcement, as well as all subsequent, were received only after shuttling to the opposite compartment and interaction with the feeder. Thus, every next sugar bead was received in the opposite feeder and the reinforced operant response was shuttling between the compartments/feeders. Prior to the first injection, mice were familiarized with the task over the course of one experimental session (5 min), and the overall number of test sessions equaled 5. The following behavioral parameters were quantified: (a) the number of transitions between compartments and (b) the total number of food reinforcements. Every session was preceded by 18 h food deprivation; at all other times, animals received food ad libitum in their homecages. After the learning session, animals were fed ad libitum in individual chambers over the course of 1 h, and the quantity of food consumed in grams was recorded. The measurement of food consumption (g per hour) and 10% sucrose preference [29] (mL per hour) was carried out in individual chambers every 3rd day over the course of the whole experiment (see Table 1).

### 2.4. Tissue Collection and Processing

To quantify the course of neurochemical/histological changes, animals in both groups were decapitated according to an experimental protocol (see Section 2.1) and the brains were removed on ice. The blocks of tissue containing brainstem and striatum were removed, dissected on ice and kept at −70 °C for biochemical measurements.

The rest of the brain was removed intact, frozen rapidly on a metal block in liquid nitrogen vapor and stored at −70 °C for histology. Cryostat slices of 20 μm thickness were made under visual control, comparing them to the stereotaxic mouse brain atlas [30] at a distance of +1.4 to −3.64 mm from Bregma in such a way that all the structures studied were present on each slice and kept frozen at −70 °C. The slices and blocks of brain tissue were processed using a number of different methods.

### 2.5. Immunohistochemistry

Brain slices were mounted on “SuperFrost” slides (Thermo Scientific, MA, USA) and fixed in 4% paraformaldehyde solution (Sigma Aldrich, MO, USA) for 15 min. Epitope retrieval was performed in a 0.1 M citric buffer solution (pH 6.0) in a steam cooker. Hellendahl staining jars with slides were placed into a steamer for 15 min. After cooling, the slides were rinsed with PBS (0.01 M, pH = 7.4, MP Biomedicals #0928103) and left in PBS containing 0.1% Triton X-100 for 1 h. Slides were incubated with: (a) rabbit polyclonal antibodies against tyrosine hydroxylase (T8700, 1:500 dilution, Sigma, MO, USA) diluted with solution containing sodium azide and bovine serum albuminfor 16 h; (b) secondary goat antibodies conjugated with CF488 fluorochrome (SAB4600045, 1:250 dilution, Sigma, MO, USA) for 2 h in a light-proof humidity chamber. Finally, slides were coverslipped in a Fluoroshield mounting medium with DAPI. Nikon Eclipse Ni-U Microscope (Nikon, Tokyo, Japan) with FITC and DAPI filter cube was used for imaging.

Images were acquired from 20 µm-thick frozen frontal slices taken with a 75-µm gap on the −2.7 to −3.64 mm level from Bregma under the same microscopic conditions, i.e., exposition time 800 ms, camera gain 1.1. On the panoramic image, the whole SNpc region and a 0.30 mm^2^ VTA located at a distance from SNpc were manually marked out, and all TH+ neuron bodies with a visible nucleus were marked using ImageJ software (National Institutes of Health, Bethesda, MD, USA). Two slices from each brain were taken for analysis.

### 2.6. Cytochrome-C-Oxidase (COX) Histochemistry

COX histochemistry was processed analogous to the procedure described in [31] and our previous work [23]. The selected regions for the analysis were anatomically defined according to the mouse brain atlas [30]. The stained mouse coronal sections corresponded to Bregma levels from +1.4 to −3.64 mm. The following structures were chosen for analysis: visual cortex (V1), motor cortex (M1), prefrontal cortex (PFC), dorsolateral striatum (DLStr), ventromedial striatum (VMStr), amygdala (Amy), thalamus (Thal), nucleus ruber (NR), substantia nigra pars reticulata (SNpr), substantia nigra pars compacta (SNpc) and dorsal (DHip) and ventral (VHip) hippocampus.

Image acquisition for COX staining intensity measurements was performed with a Cytation 5 imaging reader (BioTek Instruments Inc., Winooski, VT, USA). The mean optical density (OD) for each structure was measured manually with the use of ImageJ software for image analysis in two sections for each animal. OD data for the experimental groups were normalized to the measurements from the same regions in the control group and expressed in percentages.

### 2.7. Lipid Peroxidation and Endogenous Antioxidant System

The status of lipid peroxidation (LPO) and endogenous antioxidant system in the brainstem homogenized tissue samples were studied using Fe^2+^-induced chemiluminescence (CL) analysis in accordance with the protocol used in our previous work [24]. As in previous works, we analyzed the following reaction parameters: h, mV—rapid initial burst of CL corresponding to the level of preformed LPO products; T,c—latent period prior to further substrate oxidation, reflecting the endogenous antioxidant potential.

### 2.8. High-Performance Liquid Chromatography with Electrochemical Detection

Samples of the dorsal striatum and frontal cortex were homogenized in twenty volumes of cold 0.1 N HClO_4_ with the addition of 3,4-dihydroxybenzylamine hydrobromide (0.25 nmol/mL) as an internal standard. Samples were prepared on ice using the Shuett Homgen plus homogenizer with a glass/Teflon pestle (0.2 mm) (SchuettBiotec GmbH, Göttingen, Germany). Samples were centrifuged at 10,000× *g* for 15 min (t = 4 °C). The content of monoamines was determined in the supernatant using high-performance liquid chromatography with electrochemical detection (HPLC-ED) on a System Gold liquid chromatograph (Beckman CoulterInc., CA, USA) with an EC3000 electrochemical detector (RECIPE GmbH, Munchen, Germany) equipped with a ClinLab ECD cell, Model Sputnik, glass carbon working electrode (+0.85 V) and an Ag/AgCl reference electrode. The studied substances were separated on a reverse-phase column Nucleodur C18 Gravity, 4.6 × 250 mm, 5 μm pore diameter (Mashery-Nagel GmbH & Co. KG, Dueren, Germany).

### 2.9. Statistical Analysis

Data were analyzed using GraphPad Prism 8 (GraphPad Software, Inc., San Diego, CA, USA) software by one-way analysis of variance (ANOVA) and post-hoc Bonferroni tests. The results are expressed as the mean ± SEM, and differences were considered significant for *p* < 0.05.

## 3. Results

### 3.1. Time Course of Behavioral Changes in Rotenone-Treated Animals

#### 3.1.1. Survival, Vitality and Food Consumption

In the present study the mortality rate was quite high—27% of the mice treated with 4 mg/kg per day died during the treatment period of 5 weeks. These animals died randomly at different timepoints, demonstrated more severe symptoms than the others, and therefore, were excluded from the experimental group (*n* in experimental groups does not include these animals). The overall state of the animals was monitored using weight and foodconsumption as the main indicators. The overall mean weight of rotenone-treated animals was lower than in the control group (ANOVA F(1, 775) = 23.15, *p* < 0.001), but pairwise comparison did not show any differences at specific timepoints. Thus, there were no distinct weight changes as the result of rotenone injections. In contrast, measurements of food consumption showed both the overall difference of the rotenone-treated animals (ANOVA F(1, 144) = 43.05, *p* < 0.001) and significant decrease at specific timepoints: the 11th, 14th and 29th day of the experiment. Interestingly, the food consumption in the experimental group on other days did not differ from the control groups despite the daily rotenone injections. Measurements of sucrose preference showed the overall difference of the rotenone-treated animals (ANOVA F(1, 142) = 38.12, *p* < 0.05), but post-hoc analysis showed significant decrease only on the 17th day of the experiment.

#### 3.1.2. Motor Symptoms

**Figure 1:**
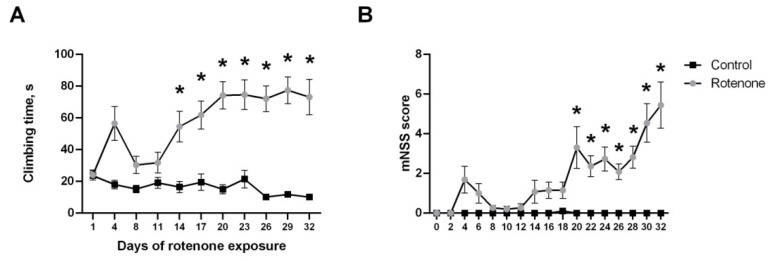
Changes in motor symptoms during 32 days of daily rotenone injections (**A**) Increase in climbing time in the gridtest depending on the day of rotenone cumulative action. (**B**) Modified Neurological Severity Score (mNSS) results depending on the day of rotenone cumulative action. * *p* < 0.05 compared to the control group at the same time point.

Motor symptoms were evaluated using both the modified neurological scale and two specific tests—catalepsy measurement and elevated grid walking. The ANOVA test revealed significant effects of rotenone treatment on all of the motor parameters measured: mNSS score (F(1, 23) = 77.24, *p* < 0.001), catalepsy time (F(1, 204) = 13.14, *p* < 0.001) and grid walking (F(1, 205) = 101.0, *p* < 0.001). According to the mNSS score, animals treated with rotenone showed significant changes in their normal behavior from day 20 onward (Figure 1B), but it should be noted that they did not demonstrate severe motor changes up to the end of the experiment—mean score in the final point was 5.44 ± 0.38 (*p* < 0.001 from control) out of a maximum of 16. A pairwise comparison of the mean catalepsy times did not show significant differences from the control group, but certain rotenone-treated animals demonstrated increased time (12–28 s) starting from day 20 onwards. Changes in the grid walking test manifested earlier (Figure 1A) and continued up to the final point of the experiment. Rotenone-treated animals showed both an increased number of paw slips from the grid on day 26–29 (*p* < 0.001 from control) and an increase in the time needed to climb from day 14 onward (Figure 1A). Some of the rotenone-treated animals stayed motionless on the grid for the whole test time by the end of the experiment (29–32 day).

#### 3.1.3. Spontaneous and Food-Motivated Motor Activity

**Figure 2:**
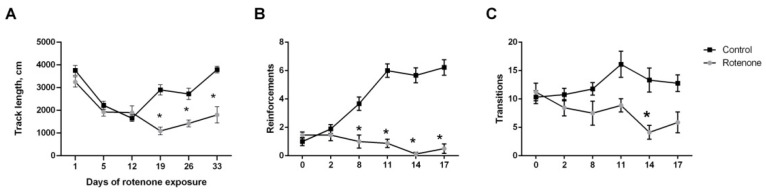
Changes in spontaneous and food-motivated motor activity over the course of experiment. (**A**) Track length in the openfield test on different days, (**B**) total number of reinforcements in the shuttlebox during 5 min tests on different days, (**C**) total number of transitions between compartments in the shuttlebox on different days. * *p* < 0.05 compared to the control group at the same time point.

In comparison with the first day, the spontaneous horizontal activity of both vehicle and rotenone-treated animals in the open field decreased up to the 12th day, but the differences between groups were not pronounced up to this point. A significant decrease in horizontal activity in the rotenone-treated group in comparison to the control group of animals started from day 19 onward (Figure 2A). On day 19, rotenone-treated animals spent 155 ± 14 s freezing in comparison to 58 ± 9 s in control animals (*p* < 0.001).

In contrast to spontaneous activity, the food-motivated activity in the shuttlebox changed earlier in experiment: the number of food reinforcements gathered by rotenone-treated animals significantly differed from the control group from day 8 onward (Figure 2B). Starting from this point, the number of reinforcements in the control group started to increase, while in the experimental animals stayed on the same level. However, these differences could rather be attributed to the lack of learning in rotenone-treated animals than to the decreased locomotion, as this parameter (transitions between compartments) was significantly different only on days 14–17 (Figure 2C). The same could be said for freezing, which also increased in the rotenone-treated group by the end of the experiment and reached 107 ± 21 s in comparison to 8 ± 2 s in control animals (*p* < 0.001) on day 17.

### 3.2. Neuropathological Effects of Rotenone

#### 3.2.1. Time Course of Histological Changes in Rotenone-Treated Animals

**Figure 3:**
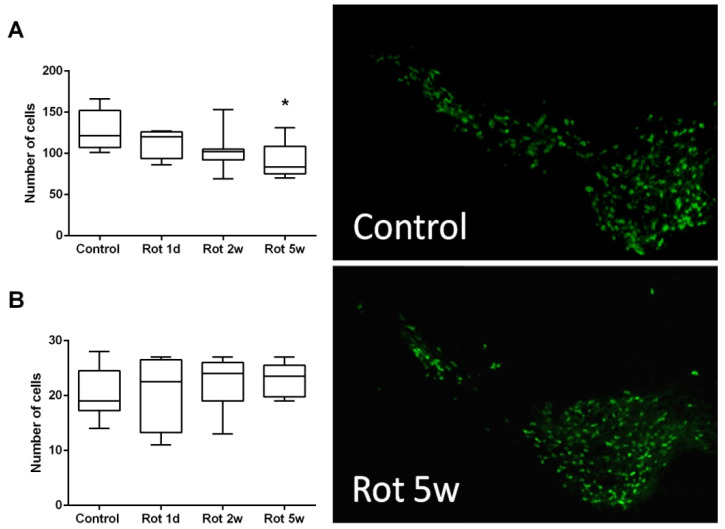
Number of TH-stained neurons in the SNpc (**A**) and VTA area (**B**), measured at different timepoints of the experiment. Reduced number of dopamine neurons in SN in mice treated with rotenone for 5 weeks in comparison to the control group (exemplary pictures right). * *p* < 0.05 compared to the control group. The pictures represent the microscopic photos with magnification 4×.

The number of TH-positive cells in SNpc decreased slightly over the course of the experiment in rotenone-treated animals, which was confirmed by ANOVA test for a linear trend (F(1, 21) = 8.9, *p* < 0.05). However, pairwise comparison of the groups showed a significant difference from control groups only in the “Rot 5 w” experimental group (Figure 3A). The decrease in the number of cells in SNpc progressed slowly and by the 5th week, the mean number of cells in rotenone-treated animals constituted 72 ± 7% of control group values, *p* < 0.05. At the same time, we were unable to show any significant changes (Figure 3B) in the number of TH-positive cells in the VTA area (F(3, 21) = 0.41, *p* = 0.75).

**Figure 4:**
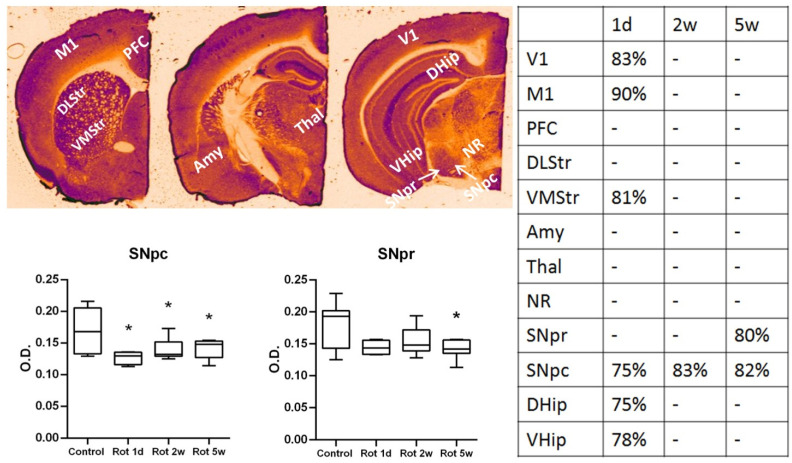
Cytochrome-C-oxidase (COX) activity, evaluated with histochemistry in experimental groups on the brain slices, containing the marked areas. Two exemplary graphs show the changes in substantia nigra pars reticulata (SNpr) and substantia nigra pars compacta (SNpc), expressed in optical density (O.D.) in comparison to control slices. The table shows all the areas and timepoints demonstrating significant changes in comparison to control slices; data are expressed in percentages from control values. * *p* < 0.05 in comparison to the control group. The colored brain slice is shown for visualization. Dark areas correspond to the high optical density after staining, and light-to low optical density (low COX activity).

The results of the comprehensive analysis of the COX activity in different areas of the mouse’s brain are summarized in the Figure 4. The analysis showed that the main differences in the COX activity are pronounced after the first day of rotenone injections. The table shows that the main regions with significantly decreased activity were SNpc, VMStr and cortical structures—V1, M1, DHip and VHip. The most affected regions were hippocampus and SNpc, showing a decrease from the control group by 25% (Figure 4). Following the course of chronic rotenone injections these acute changes of COX activity normalized and no significant differences in optical density were found in most of the areas after both the 2nd and 5th weeks. However, COX activity in substantia nigra showed a different picture—a significant decrease of COX activity in SNpc was shown in all experimental groups and in SNpr, a significant decrease was present only by the 5th week of experiment.

#### 3.2.2. Time Course of Biochemical Changes in Rotenone-Treated Animals

As summarized in Table 2, the monoamine concentration in rotenone-treated animals showed neither in the frontal cortex nor in striatum significant differences from the control group. The ANOVA with a factor of treatment has shown no differences in the concentration of monoamines studied, except for 3-MT in the striatum (F(4, 33) = 6.2, *p* < 0.001). Pairwise comparison showed that the level of 3-MT significantly decreased in rotenone-treated animals only by the 5th week of experiment (Table 2).

ANOVA with a factor of treatment also did not show any difference neither in the level of preformed lipoperoxides nor in the endogenous antioxidant system activity in the brainstem homogenates. There was merely a tendency (25% increase, *p* = 0.07) toward an increased level of lipid peroxides in rotenone-treated animals only by the 5th week of the experiment.

## 4. Discussion

The current study was designed to characterize the behavior abnormalities in C57BL/6 mice during chronic exposure to low doses of mitochondrial toxin rotenone and to describe the time course of neurodegeneration and underlying neuropathological processes both on the motor and pre-motor stages. This study is not the first to do that, as the question of differences between rotenone action on mice and rats remains current since its first use in rat models of PD progression [14]. The data on this topic remain controversial, as the effect of rotenone on mice ranges from high systemic toxicity [18,32], expressed both in mortality, behavioral and morphological changes, to the lack of visible effects in some studies [33]. Richter et al. [18] reported the decrease in motor activity in the open field test and several neurological symptoms in mice from day 15 of daily subcutaneous injections of 5 mg/kg rotenone for 45 days, but without visible neurodegeneration of dopamine neurons. In contrast, later works both with subcutaneous [21] and intragastric [22] administration of rotenone showed significant neurodegeneration and a-Syn accumulation in the SNC, DMV and ENS after 4 weeks. However, none of these works focused on the matching of behavioral and neuropathological changes over time, which is important for clarification of the mechanism of neurodegeneration in PD.

Our previous work on the topic, comparing the neurotoxicity of rotenone to that of MPTP, had shown the faster progression of PD-like symptoms and SNpc neurodegeneration, but without the decrease in dopamine in basal ganglia, characteristic to PD [24]. However, we used the outbred CD-1 mice strain, which may have significantly affected our findings. Comparison to the other studies also shows that difference in results may be partly explained by the differences in animal strains used [34]. That was the rationale behind replicating our design with continuous behavioral testing and multiple timepoints for neuropathological analysis using the standard C57BL/6 mice strain. We have also broadened the scope of the study by comparing the changes in regional brain metabolism in different brain areas over time, measured with COX histochemistry [31]. The dose of rotenone used in the current study was chosen as corresponding to the environmental exposure levels of rotenone via pesticides [17].

**Figure 5:**
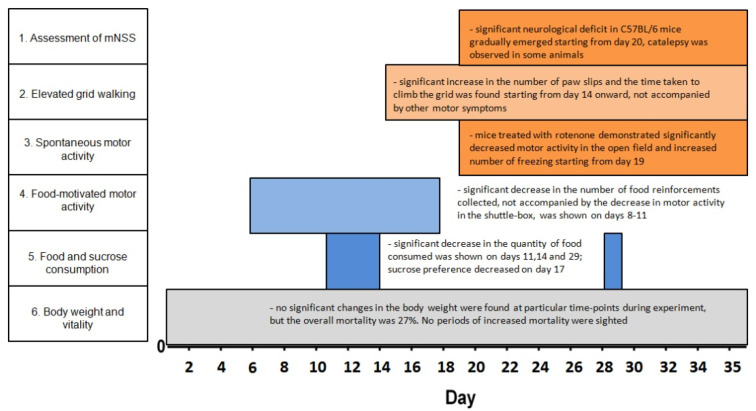
Behavioral changes in the rotenone-treated mice.

The major findings of this study include the behavioral staging of the rotenone-induced pathology in C57BL/6 mice (Figure 5) and evaluation of key pathological cascades, corresponding to these stages: SNpc neurodegeneration, lipid peroxidation and regional energy metabolism changes in the brain.

Based on the behavioral testing performed, we can roughly stage the rotenone-induced pathology into three stages: First, behavioral abnormalities in mice start from week 2 of the experiment (cumulative dose > 28 mg/kg) and include the decline in food-motivated motor activity (8 day), followed by the decrease in food consumption (11, 14 day) and sucrose consumption (17 day) in food-deprived animals. Analysis of the number of food reinforcements in the shuttle box in control animals shows that mice demonstrate the sharp increase in the number of reinforcements and transitions starting from the 3rd testing day—corresponding to a standard learning curve for the operant learning tasks [35]. In contrast, rotenone-treated mice did not show any learning progress despite unchanged motor activity: they shuttled between compartments without collecting reinforcement. This behavior can hardly be attributed to motor or activity changes, but are rather explained in terms of altered learning and motivation. The decrease in the sucrose preference, usually interpreted as anhedonia, is in line with this proposition [29]. It is known that toxins such as 6−OHDA, MPTP and rotenone are able to induce depressive-like behavior in rats [36] and we have previously shown the same disruption of learning in the shuttlebox in rats on the prodromal stage of rotenone-induced pathology [28]. To our knowledge, this is the first study to demonstrate this effect in C57BL/6 mice. Such changes are usually interpreted as depressive-like behavior alterations and associated with a disruption in VTA dopaminergic neuron functioning, a decrease in DA levels in the mesolimbic pathway [37,38] and hippocampal 5-HT reduction [36]. However, in the current study, we were unable to demonstrate significant neurodegeneration in VTA nor find any changes in the level of neurotransmitters in basal ganglia, nor in the frontal cortex. Thus, the aforementioned symptoms could rather be linked to phasic than tonic dopamine alterations [39].

The second, early motor stage starts from week 3 with subtle behavioral abnormalities in the gridwalking test (from day 14 onward) and the shuttle box (decreased number of shuttles). These changes corresponded to the cumulative dose of rotenone >56 mg/kg. The Grid walking test was used as a specific test for motor coordination and subtle motor changes in mice in early stages of PD [40], correlating with the decrease in DA level. We saw both the increased number of footfaults and increased time to ascend the grid, which could equally represent an increased immobility or a decreased motor coordination. However, there were no changes in the mNSS score at this stage, which included a similar activity test, elevated beamwalking and no DA changes. The lack of visible neurological symptoms allowed us to speculate that the disruptions in the motor system on this transition stage are limited to the skilled coordinated movements. In human studies, parkinsonian coordination problems are usually linked specifically with cortical dysfunction [41].

The visible decrease in animal motor activity can be seen only starting from week 4 (cumulative dose of rotenone >76 mg/kg) and is expressed in the mNSS score increase and pronounced immobility both in the grid walking and open-field tests. These pronounced motor symptoms became more severe towards the end of the experiment that can be seen in the temporal dynamics of mNSS score on days 20–35 (Figure 1B). The data here reproduce the other recent studies on the rotenone mice model [21,22], showing severe motor impairments after a month of chronic toxin injections. Overall, this behavioral staging adds to previous findings by showing the gradual evolution of symptoms, from motivational changes in week 2 to subtle paw coordination disruption followed by bradykinesia onset by the 4th or 5th weeks of rotenone exposure. The described symptoms are an important characteristic of the clinical manifestations of Parkinson’s disease [2,7,12] and can be studied in isolation by choosing appropriate timepoints. The study of the underlying neuropathology also requires sampling of material at multiple timepoints, which was performed in the current experiment.

We have found a decrease in the number of TH-positive cells in SNpc, the key morphological change in PD, only in the final point of the experiment, after 35 days of chronic rotenone exposure, and not in the 2-week time point that corresponds with the onset of early motor symptoms. This finding mirrors clinical data, showing that disease symptoms manifest themselves only when SNpc neurodegeneration reaches more than 50% and the dopamine level cannot be compensated by the cells left intact [1]. Moreover, the actual decline in the number of TH-positive cells was moderate, only by 28% percent, which can explain the lack of changes in the monoamine contents in both the frontal cortex and the striatum. These numbers are in agreement with previous morphological studies [16,21], but in contradiction with Richter et al.’s work, not showing significant neurodegeneration [18]. The limitation of the current study is the lack of timepoints between 2 and 5 weeks, which could show the early onset of neurodegeneration together with the manifestation of motor symptoms. However, the lack of changes in the 2-week group means that described early behavioral changes could hardly be attributed to the decline in SNpc structure and function, characteristic to PD, and require another explanation. Recent studies link the a-Syn inclusions, well described in the rotenone model, with amygdala and cortical dysfunction, which could lead to specific behavioral defects, including cognitive impairments [42].

Considering the neuropathological processes leading to SNpc neurodegeneration, the present study tends to support the position, attributing rotenone effects to mitochondrial dysfunction rather than pronounced oxidative stress in vivo [43]. We were unable to show significant changes in LPO or antioxidant potential in the brainstem homogenates in C57BL/6 in neither of the timepoints studied. However, we have shown pronounced effects of rotenone on the decrease in COX activity, usually interpreted as a decrease in mitochondrial oxidative phosphorylation and overall metabolic activity of the tissue [31]. The same as in our previous work with CD-1 mice and Wistar rats, we have shown the global non-specific effect of rotenone on different brain structures. This effect was mostly pronounced in the acute phase of rotenone action (1 day) and in cortical structures, which were previously shown to be the most metabolically active [23,44]. Particularly interesting are the transitory changes in regional metabolism in the cortex and hippocampus, which can be linked to the early symptoms observed. At the same time, the current study showed a more specific effect in C57BL/6 mice, concerning the decrease of COX activity in SNpc and SNpr in the late stages of chronic rotenone exposure. This decrease could affect basal ganglia circuitry even without pronounced changes in tonic DA levels. Moreover, these local changes are in line with the proposition that SNpc neurons, all sharing poorly myelinated, long and thin axons, are selectively vulnerable to mitochondrial dysfunction in PD [1].

Overall, the present findings add significantly to previous works on the rotenone model, not able to find SNpc neurodegeneration [18] or a decrease in the mitochondrial electron transport chain complexes activity [21], by demonstrating these changes over time and showing that C57BL/6 mice, treated with low doses of rotenone, can also be used in the modeling of different stages of PD as well as rats. However, the pathological changes, leading to the onset of early PD-like symptoms, remain to be studied.

## Figures and Tables

**Table 1:** Experimental procedures and times.

Test	Days
Neurological scale evaluation	0, 2, 4, 6, 8, 10, 12, 14, 16, 18, 20, 22, 24, 26, 28, 30, 32
Catalepsy test	0, 1, 4, 8, 11, 14, 17, 20, 23, 26, 29, 32
Elevated grid walking	0, 1, 4, 8, 11, 14, 17, 20, 23, 26, 29, 32
Open field	1, 5, 12, 19, 26, 33
Operant learning	0, 2, 8, 11, 14, 17
Sucrose, food consumption testing	0, 2, 5, 8, 11, 14, 17

**Table 2:** Level of monoamine neurotransmitters and metabolites (in nmol/g tissue) measured in homogenates of frontal cortex and striatum at different timepoints of experiment: Rot 1 d—24 h after first rotenone injection, Rot 2 w—after 2 weeks of daily rotenone injections, Rot 5 w—after 5 weeks of daily rotenone injections.

Structures		Control	Rot 1 d	Rot 2 w	Rot 5 w
Striatum	NA	1.16± 0.05	1.25 ± 0.06	1.2 ± 0.06	1.02 ± 0.04
	DA	70.6 ± 1.18	65.6 ± 0.74	68.9 ± 0.78	73.4 ± 2.01
	HVA	5.24 ± 0.11	7.21 ± 0.21	6.37 ± 0.3	10.42 ± 0.76
	DOPAC	3.01 ± 0.04	3.46 ± 0.15	4.5 ± 0.32	7.9 ± 0.78
	3-MT	3.6 ± 0.11	3.65 ± 0.15	2.7 ± 0.16	2 ± 0.125 **
	5-HT	4.25 ± 0.05	4.66 ± 0.16	4.66 ± 0.09	4.25 ± 0.09
	5-HIAA	3.24 ± 0.06	4.12 ± 0.09	5.83 ± 0.37	5.85 ± 0.33
Cortex	NA	4.39 ± 0.07	3.61 ± 0.14	4.55 ± 0.08	5.26 ± 0.15
	DA	4.1 ± 0.81	4.86 ± 1.14	1.12 ± 0.17	2.75 ± 0.44
	HVA	1.66 ± 0.06	1.84 ± 0.09	2.11 ± 0.3	3.546 ± 0.45
	3-MT	-	0.81 ± 0.34	0.17 ± 0.03	-
	5-HT	6.65 ± 0.16	6 ± 0.22	8.4 ± 0.21	6.53 ± 0.43
	5-HIAA	3.65 ± 0.16	4.96 ± 0.45	5.22 ± 0.41	4.69 ± 0.25

** *p* < 0.001 in comparison to the control group.

## Data Availability

Data available on request from the authors.

## Abbreviations

PD	Parkinson’s disease
SNpc	substantia nigra pars compacta
SNpr	substantia nigra pars reticulata
VTA	ventral tegmental area
V1	visual cortex
M1	motor cortex
PFC	prefrontal cortex
DLStr	dorsolateral striatum
VMStr	ventromedial striatum
Amy	amygdala
Thal	Thalamus
NR	nucleus ruber
DHip	dorsal hippocampus
VHip	ventral hippocampus
MPTP	1-methyl-4-phenyl-1,2,3,6-tetrahydropyridine
6-OHDA	6-hydroxydopamine
VTA	ventral tegmental area
DMSO	dimethyl sulfoxide
mNSS	modified neurological severity scores
PBS	phosphate-buffered saline
LPO	lipid peroxidation
CL	Chemiluminescence
DA	Dopamine
5-HT	Serotonin
NA	Noradrenaline
DOPAC	3,4-dihydroxyphenylacetic acid
3-MT	3-methoxytyramine
HVA	homovanillic acid
5-HIAA	5-hydroxyindoleacetic acid
HPLC-ED	high-performance liquid chromatography with electrochemical detection
TH	tyrosine hydroxylase
DAPI	4′,6-diamidino-2-phenylindole
NADH	nicotinamide adenine dinucleotide
